# Classification of Suicide Attempts through a Machine Learning Algorithm Based on Multiple Systemic Psychiatric Scales

**DOI:** 10.3389/fpsyt.2017.00192

**Published:** 2017-09-29

**Authors:** Jihoon Oh, Kyongsik Yun, Ji-Hyun Hwang, Jeong-Ho Chae

**Affiliations:** ^1^Department of Psychiatry, Seoul St. Mary’s Hospital, College of Medicine, The Catholic University of Korea, Seoul, South Korea; ^2^Computation and Neural Systems, California Institute of Technology, Pasadena, CA, United States; ^3^Bio-Inspired Technologies and Systems, Jet Propulsion Laboratory, California Institute of Technology, Pasadena, CA, United States

**Keywords:** suicide, machine learning, Psychiatric Status Rating Scales, depression, anxiety disorders

## Abstract

Classification and prediction of suicide attempts in high-risk groups is important for preventing suicide. The purpose of this study was to investigate whether the information from multiple clinical scales has classification power for identifying actual suicide attempts. Patients with depression and anxiety disorders (*N* = 573) were included, and each participant completed 31 self-report psychiatric scales and questionnaires about their history of suicide attempts. We then trained an artificial neural network classifier with 41 variables (31 psychiatric scales and 10 sociodemographic elements) and ranked the contribution of each variable for the classification of suicide attempts. To evaluate the clinical applicability of our model, we measured classification performance with top-ranked predictors. Our model had an overall accuracy of 93.7% in 1-month, 90.8% in 1-year, and 87.4% in lifetime suicide attempts detection. The area under the receiver operating characteristic curve (AUROC) was the highest for 1-month suicide attempts detection (0.93), followed by lifetime (0.89), and 1-year detection (0.87). Among all variables, the Emotion Regulation Questionnaire had the highest contribution, and the positive and negative characteristics of the scales similarly contributed to classification performance. Performance on suicide attempts classification was largely maintained when we only used the top five ranked variables for training (AUROC; 1-month, 0.75, 1-year, 0.85, lifetime suicide attempts detection, 0.87). Our findings indicate that information from self-report clinical scales can be useful for the classification of suicide attempts. Based on the reliable performance of the top five predictors alone, this machine learning approach could help clinicians identify high-risk patients in clinical settings.

## Introduction

Predicting and estimating suicidal behavior in psychiatric patients is an important and vexing clinical issue. A number of methods have been developed and tested for their ability to assess and correlate suicide attempts with various clinical factors. History of previous suicide attempts has long been considered to be a crucial factor in predicting future suicide attempts (1), but attempts history lacks predictive value as only one-third of suicide attempters have a previous history of suicide attempts (2). Clinical scales that directly assess suicidal ideation and behavior are widely used (3, 4), but they generally have a low specificity for suicide attempts as they tend to incorrectly classify low-risk suicide attempters as high-risk attempters (5).

To evaluate and understand the risk factors for suicide, the association between sociodemographic characteristics and actual suicide attempts has also investigated. In a national cohort study of Swedish adults, the presence of psychiatric disorders and chronic diseases were independent risk factors for suicide among both men and women (6). Although depressive symptoms and a family history of suicide were associated with in-patient suicide, they were inadequate in discriminating actual suicide attempters with non-attempters because of relatively low predictive value for high suicide risk categorizations (<2%) (7).

Leveraging the development and growth of various artificial intelligence techniques, recent studies have shown that machine learning approaches can be useful in estimating suicide attempts. A machine learning algorithm trained with patients’ longitudinal electronic health records reliably predicted suicidal behavior (8). Another machine learning approach showed that sociodemographic information and psychopathological factors accurately predicted actual suicide in US Army soldiers (9). Linguistic-driven models that use the text of clinical notes have also been explored, but thus far have lacked sufficient accuracy (65% or more) (10).

Although machine learning techniques yielded a high performance that could be tested in clinical practice, classifying and predicting suicide attempts remains challenging. Central problems seem to be self-reporting bias of suicide attempts and a low accuracy rate of each variable that has been used to predict suicide attempts (8). In addition to these factors, inconsistency of predictors is a major barrier.

In estimating suicide attempts with clinical scales, it is common to use one or two scales such as hopelessness (11) and suicide intention (12). This approach can show reliable performance in certain circumstances, but can easily suffer from reporting biases of the respondents. Using multiple clinical scales at the same time in estimating suicide attempts may reduce biases and increase classification performance. Furthermore, negative aspects of psychological states (e.g., depression, anxiety, and hopelessness) have been frequently used as predictors of suicide attempts, but positive aspects [e.g., life satisfaction, purpose in life (PIL), and emotion regulation] have rarely been applied as parameters in estimating suicide attempts. As the absence of positive emotions and satisfaction can contribute to suicidality (13), consideration of the degree of patients’ positivity may be needed in estimating suicide attempts.

In this study, we report a model for classifying suicide attempts in patients with mental illnesses that was built from a customized artificial neural network classifier. To derive and test this novel model, we administered 31 self-report psychiatric and psychological scales that are widely used in clinical settings, and tested whether these variables have classification power for actual suicide attempts.

## Materials and Methods

### Participants and Case Definition

A total of 760 participants who visited the Mood and Anxiety Disorder Unit of Seoul St. Mary’s Hospital, The Catholic University of Korea, between 2011 and 2017 were enrolled in the study. 730 of the 760 participants agreed to participate in the survey. Of the 730 participants, 591 (81%) visited the following outpatient clinic and 18 out of 591 (3%) did not complete the questionnaire. Thus, survey data from 573 participants were used to train neural networks.

The characteristics of participants are presented in Table 1. Most of the participants (45.9%) had a depressive disorder as a primary diagnosis, with an anxiety disorder being the second most common illness. All diagnoses were based on the Diagnostic and Statistical Manual of Mental Disorders-IV (14).

**Table 1:** Sociodemographic characteristics of participants, with average scores of psychiatric scales.

Characteristics (categorical variables)	*N*	%	Characteristics (continuous variables)	Mean	SD
Gender					
Male	267	46.6	Age (years)	35.6	13.2
Female	306	53.4	Pain (NRS score)	4.8	2.3
Religion			Psychiatric scales total score		
Catholic	138	24.1	ERQ	38.7	10.9
Christian	139	24.3	ARS	47.9	14.2
Buddhism	58	10.1	SWLS	14.8	7.5
Others	29	5.1	SAI	65.8	10.9
None	209	36.5	ASI	79.5	29.2
			SHS	24.4	10.7
Marriage status			SSI	10.0	8.2
Single	298	52.0	LOT-R	13.7	5.0
Married	216	37.7	SCL	58.4	19.0
Divorced	18	3.1	BIS*	48.7	6.7
Widowed	3	0.5	PWBS	138.6	11.3
Others	38	6.6	CD-RISC	45.5	19.1
			PANAS-N	20.3	10.3
Residence			FACIT	21.0	10.4
Urban area	532	92.8	PIL	79.5	8.7
Others	41	7.2	CERQ	102.5	17.5
			SDHS	9.4	4.5
Employment status			BIS^†^	22.4	3.6
Employed	166	28.9	PCCTS	3.4	4.7
Unemployed	132	23.0	BHS	8.9	6.4
Housewife	115	20.0	IIP	66.8	25.6
Student	108	18.8	CTQ	54.9	14.4
Others	52	9.1	LEC	64.0	13.3
			BDI	25.9	12.2
Pain			FSSQ	38.4	12.2
Yes	401	70.0	BAS	34.5	6.2
No	159	27.7	GQ-6	27.1	6.9
Others	13	2.3	RRS	62.0	13.7
			PSS	26.8	6.6
Diagnosis			PANAS-P	8.5	6.3
Depressive disorder	263	45.9	TAI	61.1	12.0
Anxiety disorder	172	30.0			
Comorbid of depressive and anxiety disorders	53	9.2			
OCD	28	4.9			
PTSD	21	3.7			
Somatization disorder	8	1.4			
Bipolar disorder	3	0.5			
Insomnia disorder	1	0.2			
Others	24	4.2			

Abbreviations for Psychiatric Scales: ERQ, Emotion Regulation Questionnaire; ARS, Anger Rumination Scale; SWLS, Satisfaction with Life Scale; SAI, State Anxiety Inventory; ASI, Anxiety Sensitivity index; SHS, State Hope Scale; SSI, Scale for Suicide Ideation; LOT-R, Life Orientation Test-Revised; SCL, Symptom Check List; BIS*, Barratt Impulsiveness Scale; PWBS, Psychological Well-Being Scale; CD-RISC, Connor–Davidson Resilience Scale; PANAS-N, Positive Affect and Negative Affect Schedule-Negative; FACIT, Functional Assessment of Chronic Illness Therapy; PIL, purpose in life; CERQ, Cognitive Emotion Regulation Questionnaire; SDHS, Short Depression-Happiness Scale; BIS^†^, Behavioral Inhibition System scale; PCCTS, Parents-Child Conflict Tactics Scale; BHS, Beck Hopelessness Scale; IIP, Inventory of Interpersonal Problems; CTQ, Childhood Trauma Questionnaire; LEC, Life Events Checklist; BDI, Beck Depression Inventory; FSSQ, Functional Social Support Questionnaire; BAS, Behavioral Activation System scale; GQ-6, Gratitude Questionnaire-Six Item; RRS, Rumination Response Scale; PSS, Perceived Stress Scale; PANAS-P, Positive Affect and Negative Affect Schedule-Positive; TAI, Trait Anxiety Inventory. Abbreviations for Psychiatric Diseases and Other Variables: OCD, obsessive–compulsive disorder; PTSD, posttraumatic stress disorder; NRS, Numeric Rating Scale.

Participants could choose to complete either a booklet that contained all relevant questionnaires or an online website survey that was similar to the booklet. Participants were asked to complete the survey by the next outpatient visit, and the average return period for both the booklet and online surveys was about 1 week. If all survey contents were completed, the data of the participant were included in further analyses. To estimate the reliability and internal consistency of the questionnaires, we measured the Cronbach’s α for the 31-item scale, obtaining a value of 0.78 (SD = 0.20). All participants were informed of the emergency phone number when they were distressed during the response to the survey.

A suicide attempt was defined according to the following three questions: (1) “Have you ever attempted suicide in your lifetime?” (2) “Within the past year, have you attempted suicide?” (3) “Within the past month, have you attempted suicide?” If the participant answered “yes” to any of the above questions, they were asked to write down the number and the method of their attempts. Among the 573 participants, 163 (28.4%) had a history of suicide attempts in their lifetime, of which 68 (11.9%) were in the past year and 39 (6.8%) in the previous month (Table 2). All subjects who participated in this study provided written informed consent, and the study was approved by the Institutional Review Board of the Ethics Committee of Seoul St. Mary’s Hospital at The Catholic University of Korea (KC09FZZZ0211).

**Table 2:** Confusion matrix and classification scores in each predictive model.

	1-month suicidality	1-year suicidality	Lifetime suicidality
True-positive	5	23	127
True-negative	532	497	374
False-positive	2	8	36
False-negative	34	45	36
Accuracy, %	93.7	90.8	87.4
Specificity, %	99.6	98.4	91.2
Sensitivity, %	12.8	33.8	77.9

### Measurement of Psychiatric Rating Scales

To measure the subjective symptoms and individual characteristics of the participants, we adopted 31 self-report rating scales that are widely used in psychiatry and clinical psychology (a list of all scales is presented in Figure 2). Scales included assessments that are known to be suitable for measuring depression and anxiety symptoms, such as the Beck Depression Inventory (BDI) and State-Trait Anxiety Inventory (15, 16). Scales for measuring cognitive emotion regulation strategies were also selected (e.g., Connor–Davidson Resilience Scale and Cognitive Emotion Regulation Questionnaire), as these traits have been significantly correlated with resilience in patients with depression and anxiety disorders (17, 18). As it has been suggested that the lack of positive expectancies are also related to degree of depression, we included scales that measure one’s positivity and attitude toward their life [e.g., Life Orientation Test-Revised (LOT-R) and PIL (19)].

Each participant completed the presented form, which consisted of 31 rating scales and 3 questionnaires about history of suicide attempts. The total scores of each scale were used as predictors, and suicide attempts (yes = 1, no = 0) was set as the primary outcome. Then, we divided the 31 scales into two categories based on characteristics of positivity and negativity. If the scale mainly measures positive aspects of emotion or a psychological state (e.g., optimism in LOT-R), it was classified as a positive scale. The Anxiety Sensitivity Index (ASI) and Childhood Trauma Questionnaire belonged to the negative scale category, as they measure the degree of anxiety and traumatic experiences of childhood, respectively. 15 scales were assigned to the positive category (green text in Figure 2), and 16 to the negative category (red text in Figure 2). Figure 2 details the classification of positive and negative scale; green text represents the positive category, and red text denotes the negative category.

To compute the relative contribution of each variable, we removed the variable and computed the classification performance in its absence (40 variables). Then, we ranked the variables from the highest cross entropy to the lowest. High cross entropy indicates that the classification performance was highly disrupted because of the removal of the variable, meaning that the variable highly contributed to the classification performance.

### Model Development and Validation

Forty-one variables (31 psychiatric scales and 10 sociodemographic variables) were used as the inputs for an artificial neural network that consisted of 1 hidden layer with 41 neurons (Figure S1 in Supplementary Material). Two parameters (presence or absence of a suicide attempts) were set as the output. Data were randomly divided into three sets (70% for training, 15% for validation, and 15% for test), and the scaled conjugate gradient method was used for training (20, 21). Training automatically stopped when validation reached the minimum cross entropy, and the performance of each variable was measured by the value of cross entropy (Figure S2 in Supplementary Material).

## Results

### Classification of Suicide Attempts with All Variables

**Figure 1:**
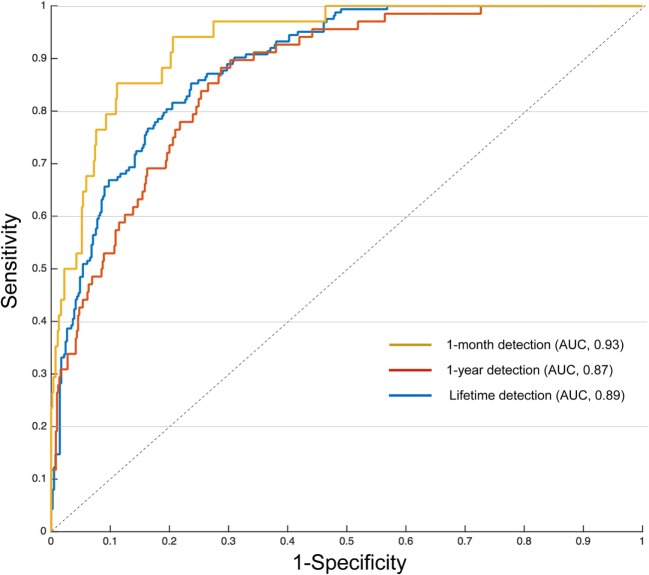
Receiver operating characteristics curves and area under the curve (AUC) for classifying suicide attempts with 41 predictors.

The artificial neural network pattern classifier trained with 31 psychiatric rating scales and 10 sociodemographic elements showed a reliable performance in classifying suicide attempts (Figure 1). The overall accuracy rate was the highest for 1-month suicide attempts detection (93.7%), followed by 1-year and lifetime detection (90.8 and 87.4%, respectively). The area under the receiver operating characteristic curve (AUROC) was also highest for 1-month suicide attempts detection (0.93), followed by lifetime (0.89) and 1-year detection (0.87). The confusion matrix and classification scores of each predictive model are presented in Table 2. Our model had high specificity for the detection of suicide attempts (99.6% in 1-month, 98.4% in 1-year, and 91.2% in lifetime suicide attempts), but the sensitivity was relatively low except in the case of lifetime suicide attempts detection (77.9%) (Table 2).

### Contribution of Each Variable in Classifying Suicide Attempts

**Figure 2:**
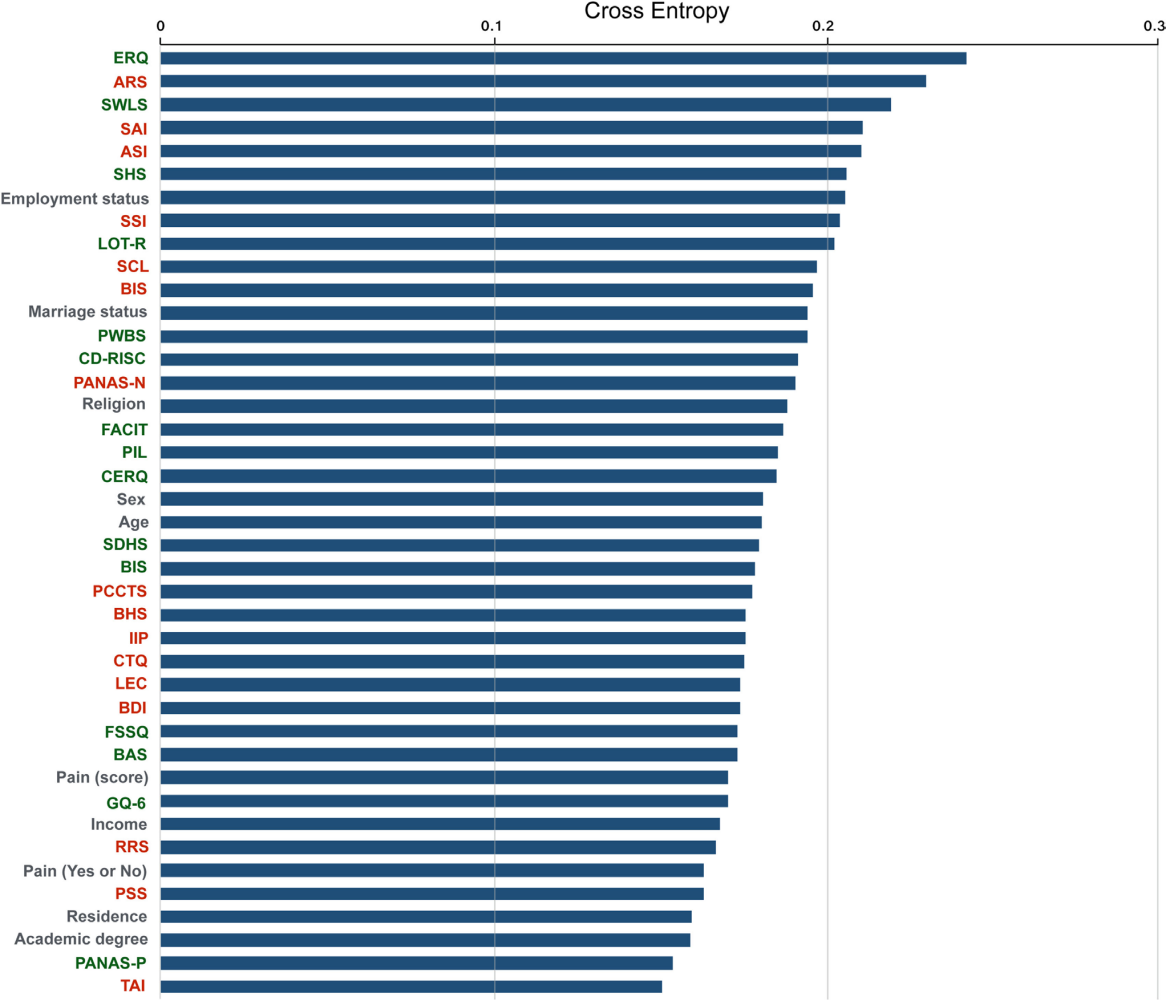
Contribution ranking for classifying suicide attempts with all predictors. The text colored in green represents scales that mainly measure positivity, whereas the text colored in red represent scales primarily measuring negativity. The gray text denotes the sociodemographic data.

Performance analysis revealed the contribution of each variable in the classification of suicide attempts (Figure 2). Among 41 variables, the Emotion Regulation Questionnaire (ERQ) had the highest contribution, followed by the Anger Rumination Scale (ARS) and the Satisfaction with Life Scale (SWLS). Although the Scale for Suicide Ideation (SSI) directly measures the wish to die and frequency of suicide ideation (4), it ranked eighth out of all scales for predicting actual suicide attempts. Among the sociodemographic information, the status of employment occupied the largest contribution in the classification of suicide attempts, followed by marriage status and religious beliefs.

### Effects of Scale Characteristics on Classification Performance

To investigate whether the characteristics of psychiatric rating scales were related to the classification of suicidality, we independently trained the artificial neural network with positive or negative scale categories and 10 sociodemographic elements.

**Figure 3:**
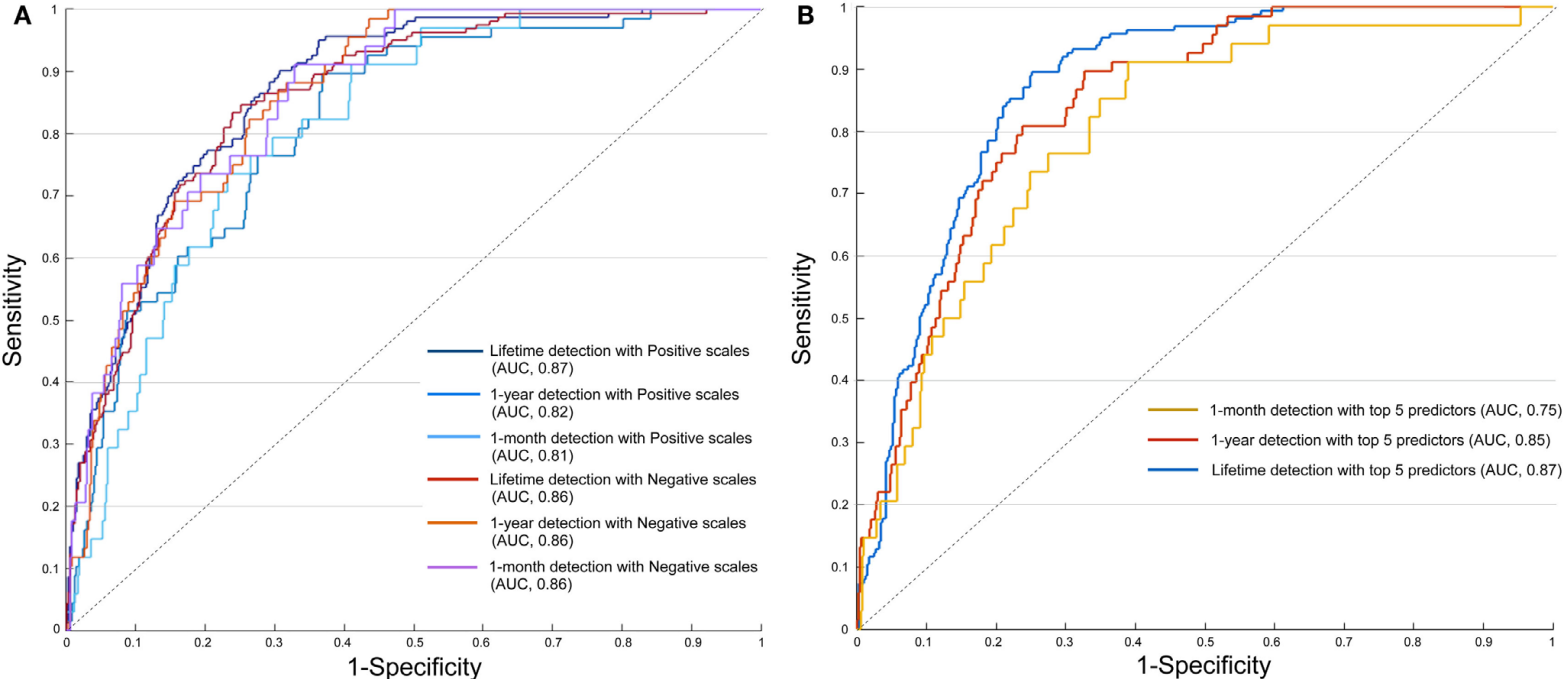
Receiver operating characteristics curves and area under the curve (AUC) for classifying suicide attempts with different categories of clinical scales **(A)** and with the top five ranked variables **(B)**.

The classification performances of each model are presented in Figure 3A. The overall accuracy rate and AUROC were not largely different between the two models. AUROC for the lifetime suicide attempts detection was slightly higher in the model trained with positive scales than in the model trained with negative scales (positive = 0.87, negative = 0.86), but the accuracy of the two groups was similar (positive = 79.9%, negative = 79.9%). AUROC for 1-year and 1-month detection was lower in the model with positive scales than negative scales (positive vs. negative; 1-month, 0.82 vs. 0.86, 1-year, 0.81 vs. 0.86). However, the overall accuracy of the two categories was not largely different, either in the 1-month or 1-year suicide attempts detection (model with positive scales vs. model with negative scales; 1-month, 92.8 vs. 92.8%, 1-year, 87.6 vs. 87.8%).

### Classification of Suicide Attempts with the Top Five Ranked Variables

To evaluate the applicability of our findings in the clinical setting, we examined whether the model could classify suicide attempts with a small number of predictors. The artificial neural network was newly trained with the top five ranked variables (ERQ, ARS, SWLS, SAI, and ASI), and none of the sociodemographic information was used. Our results showed that the classification performance was relatively preserved for detection of lifetime (AUROC and accuracy rate; 0.87 and 79.6%), 1-year (0.85 and 89.2%), and 1-month detection (0.75 and 92.7%) of suicide attempts. Adding sociodemographic information to the model trained with the top five ranked variables improved the classification performance of 1-month detection (AUROC from 0.75 to 0.81; Figure S3 in Supplementary Material). However, there was no AUROC change in 1-year and lifetime detection (1-year, 0.85; lifetime, 0.87).

## Discussion

Using a customized artificial neural network classifier, we showed that synthetic information from multiple self-report clinical scales and sociodemographic data well classified actual suicide attempts. A neural network with 41 parameters could identify retrospective lifetime suicide attempts with an AUROC of 0.89. With only five parameters, the model estimated lifetime suicide attempts with an AUROC of 0.87. Thus, we showed that this trained neural network with multiple self-report clinical scales was effective in correlating clinical scales and patients’ history of suicide attempts. Recent machine learning algorithms predicting suicidal behavior with electronic health records (8) or with sociodemographic information (9) showed a similar performance (AUROC; 0.83–0.85). However, direct comparison of our study to these previous reports should be done with caution, as we estimated retrospective suicide attempts rather than future ones.

The proposed model in this study had 41 predictors, but the characteristics of these variables were different from previous models for suicide attempts detection. Although clinical features of affective disorders (e.g., loss of interest and hopelessness) are well-known predictors of suicide (11, 22), these parameters were not used in our analysis. Instead, we included factors relevant to suicide propensity by using the total score on a battery of psychiatric and cognitive clinical scales (e.g., Beck Hopelessness Scale and BDI). Information on the participants’ diagnoses or substance use were also not used in training, though the lifetime risk for suicide has been shown to significantly vary between psychiatric illnesses (12, 23), and alcohol abuse was shown to be an independent predictor of suicide (9). Participant-reported subjective symptoms and psychological states were the main variables in our analysis, and the total number of variables was much less than in previous studies; previously, one study used 73–122 predictors (8), and another used over 100 (9). With about half the number of predictors, we showed that classification performance of suicide attempts could achieve a similar or higher AUROC value (0.89–0.93 with 41 predictors), though our study retrospectively detected suicide attempts, not future attempts.

Our results indicating high accuracy of our model in suicide attempts classification might owe to the direct measurement of suicide ideation, because we used the SSI (4) as a predictor. The SSI consists of 19 items that directly measures the “wish to live,” “wish to die,” and “frequency of suicide ideation.” Although a previous study showed that SSI was a significant measurement in detecting eventual suicide (24), it was only the eighth highest contributor in our model (Figure 2). Rather than the direct assessment of suicide ideation, indirect measures such as the ERQ, ARS, and SWLS were better predictors of actual suicide attempts. Furthermore, even without information from SSI, our model successfully classified suicide and non-suicide attempters (Figure 3B). Thus, the high accuracy rate and high AUROC value might be related to the subjective information of multiple scales, rather than direct information on suicide ideation.

While a number of studies have found that negative psychological states, such as hopelessness (11), anxiety (25), and depression (26) are related to suicidality, few studies have identified the role of positivity on suicide attempts. Our results showed that there were minimal differences between positive clinical scales and negative ones in detecting actual suicidal attempts. In addition, the best predictor of suicide attempts was the ERQ (27), which does not seem related to suicidality. This 10-item scale was designed to measure participants’ tendencies to regulate their emotions with two positive strategies—cognitive reappraisal and expressive suppression. Individuals with dysfunction in the neural circuitry of emotion regulation are at risk for violence and aggression (28), and deficits in emotion regulation are known to be associated with self-harm behavior (29). As the main outcome of this study was “actual suicide attempts” rather than “eventual suicide,” the status of emotion regulation might have made an important contribution to our findings. These results suggest that both lack of positive aspects of psychological states and excess negative emotions might similarly contribute to the detection of suicide attempts.

Our model with 41 predictors had a reliable performance in classifying 1-month, 1-year, and lifetime suicide attempts. However, it is difficult to measure over 30 self-report scales in the outpatient setting. Thus, it is notable that our model with only five predictors made a relatively accurate classification of suicide attempts with the exception of 1-month suicide attempts detection (Figure 3B). This result is somewhat interesting, as we did not include any sociodemographic information and only used the total score from five self-report psychiatric scales. As the performance of the neural network generally increases with number of predictors and sample size (30), we would expect weaker performance with 5 variables than with all 41 variables.

Several limitations should be considered in interpreting our results. First, we note that the sensitivity of 1-month and 1-year suicide attempts detection was fairly low in our model (12.8% in 1-month detection, 33.8% in 1-year detection). These results might be due to the small number of actual suicide attempts (*N*; 1-month = 39, 1-year = 68), as the classification power can be limited by training sample size (31). The lifetime suicide attempts category (163 samples) of our model showed improved performance (sensitivity = 77.9%) vs. 1-month and 1-year suicide attempts. Second, there was heterogeneity in the diagnosis of psychiatric illnesses. As patients with depression and anxiety disorders were similarly distributed in our sample, it is difficult to apply our results to specific illness groups. Third, as the inner workings of machine learning algorithms act like a “black box,” it is more difficult to interpret the meaning of this model compared to classical approaches (e.g., Bayesian modeling) (8). Fourth, administering a large number of questionnaires to a psychologically sensitive population raises ethical concerns. Although we provided a choice of response type (offline or online survey) and did not severely restrict the reply period of questionnaires, responding to all of 31 self-report questionnaires can be quite burdensome, especially to in-patients.

In conclusion, our findings suggest that systemic information reported on multiple self-report psychiatric scales can accurately classify suicide attempts. Scales that measure positive psychological state yielded comparable and even greater classification performance than scales with negative psychological states. Our model for suicide attempts detection with only five self-report scales has practical implications for the screening and estimation of suicide risk in clinical settings. This machine learning approach can be used in precision medicine in that it can assess and estimate one’s suicide attempts based on individual psychiatric and psychological scales.

## Ethics Statement

This study was carried out in accordance with the recommendations of Institutional Review Board of the Ethics Committee of Seoul St. Mary’s Hospital at The Catholic University of Korea with written informed consent from all subjects. All subjects gave written informed consent in accordance with the Declaration of Helsinki. The protocol was approved by the Institutional Review Board of the Ethics Committee of Seoul St. Mary’s Hospital at The Catholic University of Korea.

## Author Contributions

JO and J-HC conceived the idea and designed the study. J-HH and J-HC acquired the data. JO, KY, and J-HC analyzed, interpreted the data, and drafted the manuscript. All authors commented and supervised on the manuscript and approved the final version of the manuscript.

## Conflict of Interest Statement

The authors declare that the research was conducted in the absence of any commercial or financial relationships that could be construed as a potential conflict of interest.

## Supplementary Material

Figure S1 Neural network structure with one hidden layer.

Figure S2 Cross entropy change of neural network classifier according to epoch.

Figure S3 Receiver operating characteristics curves and area under the curve (AUC) for classifying suicide attempts with top 5 predictors and 10 sociodemographic information.
