# Supplementary figures and images for: Classification of Suicide Attempts through a Machine Learning Algorithm Based on Multiple Systemic Psychiatric Scales
Source: Front Psychiatry. 2017 Sep 29;8:192. doi: 10.3389/fpsyt.2017.00192 (PMC5632514; doi:10.3389/fpsyt.2017.00192)

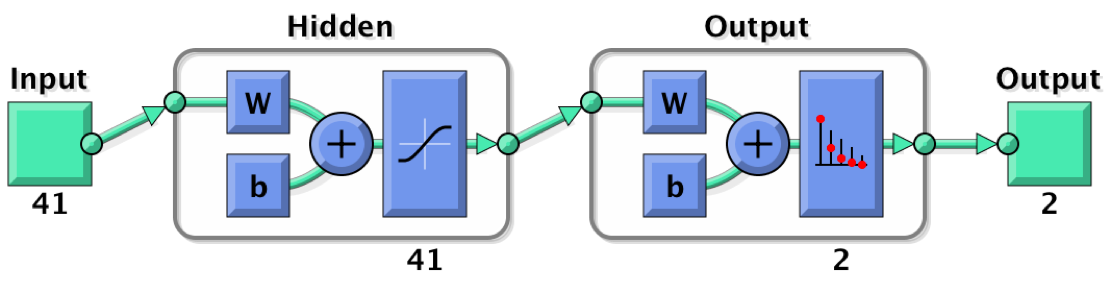

Supplement: Figure S1 — Neural network structure with one hidden layer. [file image_1.png]

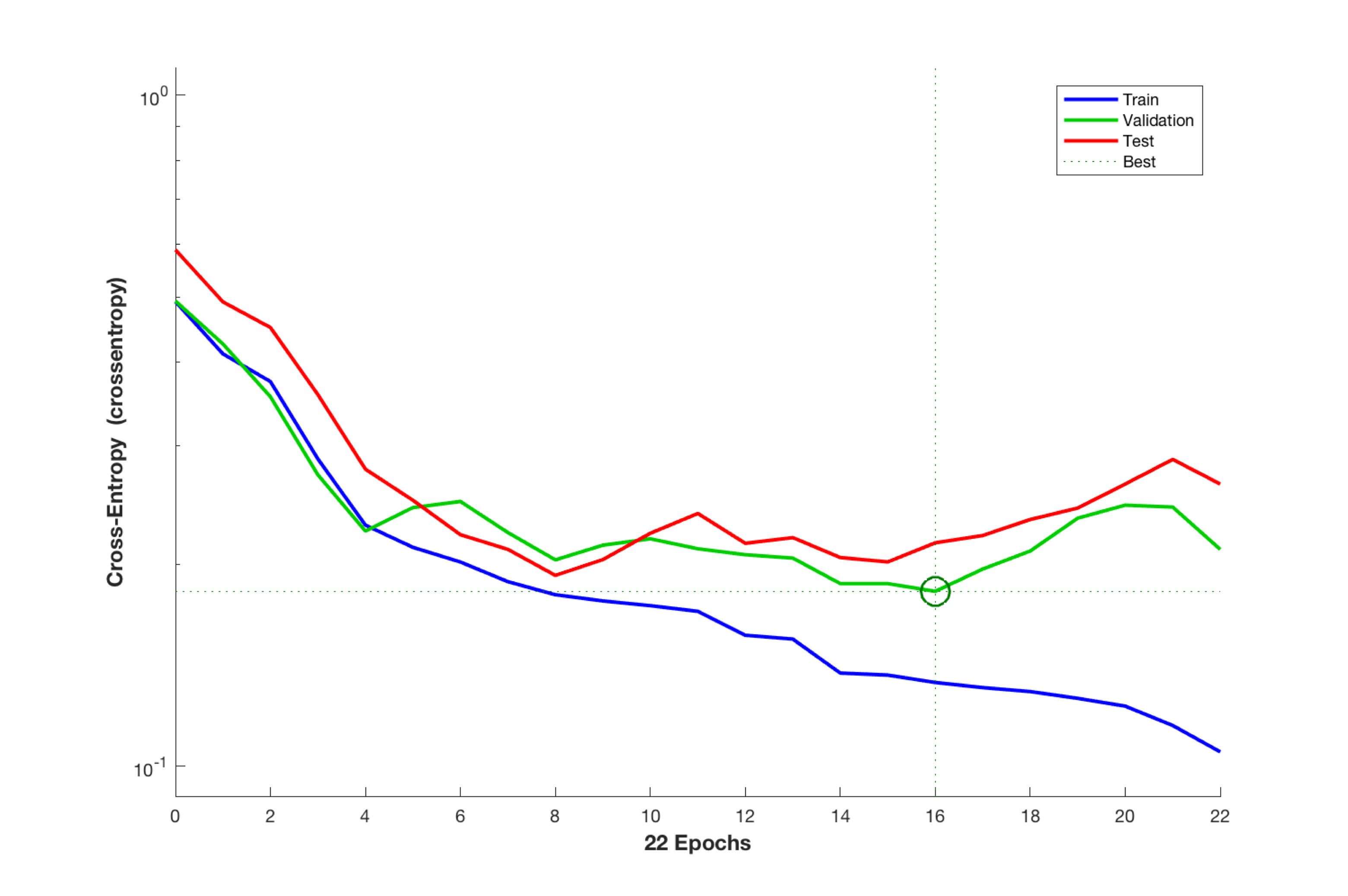

Supplement: Figure S2 — Cross entropy change of neural network classifier according to epoch. [file image_2.png]

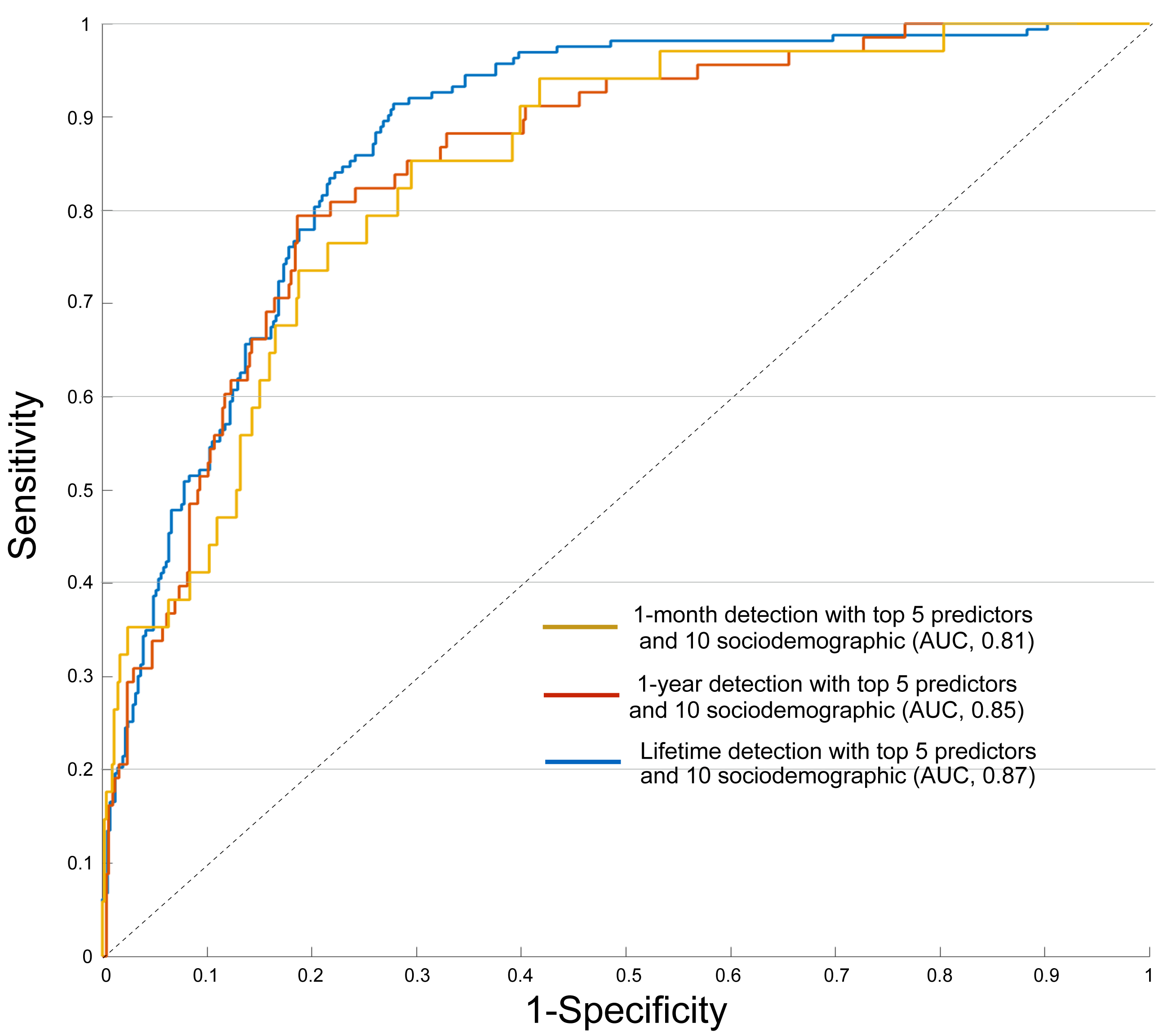

Supplement: Figure S3 — Receiver operating characteristics curves and area under the curve (AUC) for classifying suicide attempts with top 5 predictors and 10 sociodemographic information. [file image_3.tif]
